# Intersecting dimensions: advanced analytical approach to school climate and injury prevention in health policy

**DOI:** 10.1186/s13584-024-00659-z

**Published:** 2024-12-09

**Authors:** Anna Khalemsky, Eli Jaffe, Michael Khalemsky

**Affiliations:** 1 Management Department, Hadassah Academic College, Jerusalem, Israel; 2 MDA: Magen David Adom, Jerusalem, Israel

**Keywords:** School climate, Injury prevention, Primary schools, Taxonomy, Cluster analysis

## Abstract

**Background:**

Child safety in schools is paramount for decision-makers globally, with a focus on ensuring children return home safely. However, the prevalent issue of injuries across educational systems demands a comprehensive investigation into their causes, incorporating interdisciplinary perspectives and social dynamics, to develop effective prevention strategies. The objective of this study is to comprehensively analyze the factors contributing to school-related injuries and examine the impact of school climate on student safety. By employing advanced data analysis techniques, the research aims to develop targeted, effective strategies to enhance child safety in educational settings. This research aims to develop a multidimensional taxonomy to understand child injuries in elementary schools better, enhancing precision in decision-making.

**Methods:**

Data from 363 Israeli primary schools and 10,855 school injuries attended to by MDA, the principal EMS provider, were analyzed. The study utilized a two-level taxonomy, employing clustering methodology to classify schools into distinct climate "patterns," with each pattern further delineating school injury characteristics into sub-patterns. The chosen method proved effective in revealing nuanced relationships between school injuries and climate characteristics.

**Results:**

Analysis revealed five school climate clusters, ranging from "good" to "bad," each exhibiting two homogeneous sub-clusters of school injuries. Schools with a "positive" climate witnessed boys predominantly experiencing head injuries during breaks, while girls often sustained limb injuries from playing in corridors. Conversely, within the "negative" climate cluster, subgroups emerged based on injury nature, whether linked to playing or falling from a height.

**Conclusion:**

The research delineates a nuanced association between school climate and injury rates, emphasizing the necessity for sophisticated analytical techniques beyond conventional methodologies. Utilizing a diverse dataset from various disciplines, the study highlights the multidimensional aspects of school health. The developed taxonomy reveals the complex dynamics within school environments, advocating for customized health policies to mitigate injuries. Critical findings prompt a reevaluation of established assumptions about the school climate-injury relationship, informing strategic policymaking. For example, it suggests collaboration to enhance school safety through targeted, gender-sensitive interventions and improvements. Integrating different data sources offers a holistic understanding crucial for effective health policy formulation in educational contexts.

## Background

Ensuring safety for children aged 6–12 is principal to decision-makers across sectors [2, 23, 34, 35]. Above all, the system's commitment is to the parents entrusting their children to it, anticipating their safe return at day's end. The main challenge lies in the widespread occurrence of injuries, which is not limited to a single school, but permeates the entire education system [12]. To effectively address this issue, it's imperative to identify the causes of these injuries through a holistic, interdisciplinary approach, taking into account social dynamics and other contributing factors [8]. The World Health Organization (WHO) considers school injuries as a worldwide problem affecting both developed and developing countries [26, 38]. To address this issue, collaborative efforts among authorities, agencies, schools, healthcare providers, and law enforcement should be established to form a proactive support system [14, 32]. Collecting data on injuries and school climate enables the development of tailored and precise preventive actions [32]. As a result, data-driven improvement policies are strongly recommended [6].

Child intentional and unintentional injuries stem from a complex interplay of factors such as physical conditions, supervision, home environment, and local authority engagement [30, 32]. These elements don't act independently; they interact and influence each other, forming a complicated web of connections rather than a linear cause-and-effect scenario [30]. According to Kraus, children spend up to 50% of their time in school, and the risk of school-related accidents is high, especially during sports activities [19, 33]. Jaffe et al. [18] found that games are the most prominent injury cause, followed by slipping, and a relatively small part of school injuries occurred during sports activities.

Student vulnerability was found to be related to the school climate and physical risks at school [11, 21]. The school climate, measured using scientifically validated questionnaires, profoundly impacts children's safety [7, 34]. There are three dimensions of the school climate mentioned in the literature: physical, social and academic [20]. The interplay between physical aspects and supervision can influence the climate positively or negatively, underscoring the intricate connection between these elements in shaping a secure educational environment [13]. The positive school climate metrics are strongly associated with safety, healthy relationships, bullying prevention measures, engaged learning, and school improvement efforts [34, 36]. Negative school climate metrics, as expected, are strongly associated with injuries, violence, high dropout rates, poor learning performance, and low motivation, both for children and teaching staff [20, 22, 37]. There is a constant mutual interaction between children, teachers, and parents in the context of the creation of a safe environment [5, 25, 27, 31]. Mitchell et al. revealed in relation to "school climate patterns," classroom-level elements exhibited a stronger correlation with teachers' outlook on the climate, while school-level factors demonstrated a closer connection with students' perspectives [28].

Different aspects regarding school injuries and school climate exist in one shared universe: they intricately interact and influence each other. Enhancing the school climate, improving physical conditions, ensuring proper supervision, nurturing the home environment, and involving local authorities are all vital for child safety [29]. It is crucial to recognize the complexity of assessing and utilizing information related to school climate, vulnerability, and decision-making [16].

The comprehensive research must address multiple dimensions of the problem. It must integrate data from various sources [8] and use advanced data analysis techniques, such as data mining, machine learning, and statistical modeling. It can help uncover patterns and relationships that might not be immediately apparent. It's essential to understand the local context in which the data is collected. Different schools, communities, and regions might have unique challenges and strengths [1, 17]. The research must consider the combination of qualitative and quantitative methods to interpret the results [24]. Finally, the vision must be interdisciplinary and comprehensive to bridge the gap between different coordinate systems, aligning the motivations and goals of different decision-making authorities [8].

In our research, we use the cluster analysis methodology that helps to group cases into homogeneous groups without defining the potential classes [15]. In the context of school injuries and/or school climate, a "class" refers to a category or group into which schools are classified or grouped based on certain attributes or features. Cluster analysis allows us to create artificial classes that we later interpret. If such segmentation eventually leads to logically functional conclusions, this analytical process will have a chance to stimulate decision-making [4]. The clustering process was conducted in two stages, treating the resulting clusters as latent classes at each step. First, all cases were segmented based on the cluster "membership" of school climate. Then, within each identified cluster, further clustering was performed based on the characteristics of the injuries [3]. Two-level taxonomy, presented in this study, uses clustering methodology in a novel effective way. The combined clusters that are identified in the present study will lead to better understanding and more effective multi-level decision-making.

## Methods

### Data sources

The research is based on two independent datasets. The first dataset consists of 10,855 cases of school injuries in children in Israeli primary schools from 2013 to 2019. The dataset was provided by MDA (Israeli EMS service). MDA is a primary medical services provider and provides medical services in all schools and kindergartens. Due to the need to protect patient privacy, no personal details about the children were obtained. Each case was assigned a unique identification number, making it impossible to determine if the same child was injured multiple times. The second dataset with information on school climate includes 363 schools provided by the Israeli Ministry of Education. We have matched the school climate information and injury cases that happened in the same school and the same year.

### Design

The ***school injury*** information is gathered according to MDA reports. Five variables were used: (1) Event place (class, corridor, sports ground, sports hall, stairs, yard, other); (2) Event term (before-after school, break, lesson, sports class, other); (3) Anatomic place (hand, foot, head, other); (4) Injury cause (game, falling from height, slipping, violence, other); (5) Injury type (medium/deep incision, superficial incision, light local burn, rubbing tear, trauma, other). The ***school climate*** information is gathered from the Israeli Ministry of Education reports. Feature selection analysis was conducted using Principal Component Analysis, using IBM SPSS (version 28). From 13 items in the original questionnaire, the following six items remained: (1) Teachers and students' closeness; (2) Efforts involved; (3) Violence cases; (4) Teachers' satisfaction; (5) Parents involvement; (6) General positive feeling (GPF). All measurements are scaled 0–100 except the number of violence cases. The variables selected for further analysis were those included in the first two components, which together accounted for 78% of the total explained variance. Only the climate variables with an absolute correlation greater than 0.5 with one of the components were retained for subsequent analysis. Additional variables included in the analyses, collected from both datasets: (1) Exact date and hour of injury event; (2) Age group; (3) Number of regular classes in school; (4) Number of special education classes in school; (5) Average number of students in regular classes; (6) Average number of students in special education classes; (7) Gender.

### Data integration

The integration process between the two databases involved several steps:

(1) School climate data was verified for each institution (elementary, middle, and high schools) as reported in the education system, with each school identified by a unique institution number. (2) The child injury database spanned seven years, from 2013 to 2019. Consequently, data from the same period was also used from the Ministry of Education's database. (3) Some schools had complete data for each of the seven years, allowing a direct match between a child's injury in a particular year and the school's climate data for that same year. However, in cases where schools lacked data for specific years, older data from the same institution was used when no current information was available for that year.

### Data analysis

**Fig. 1:**
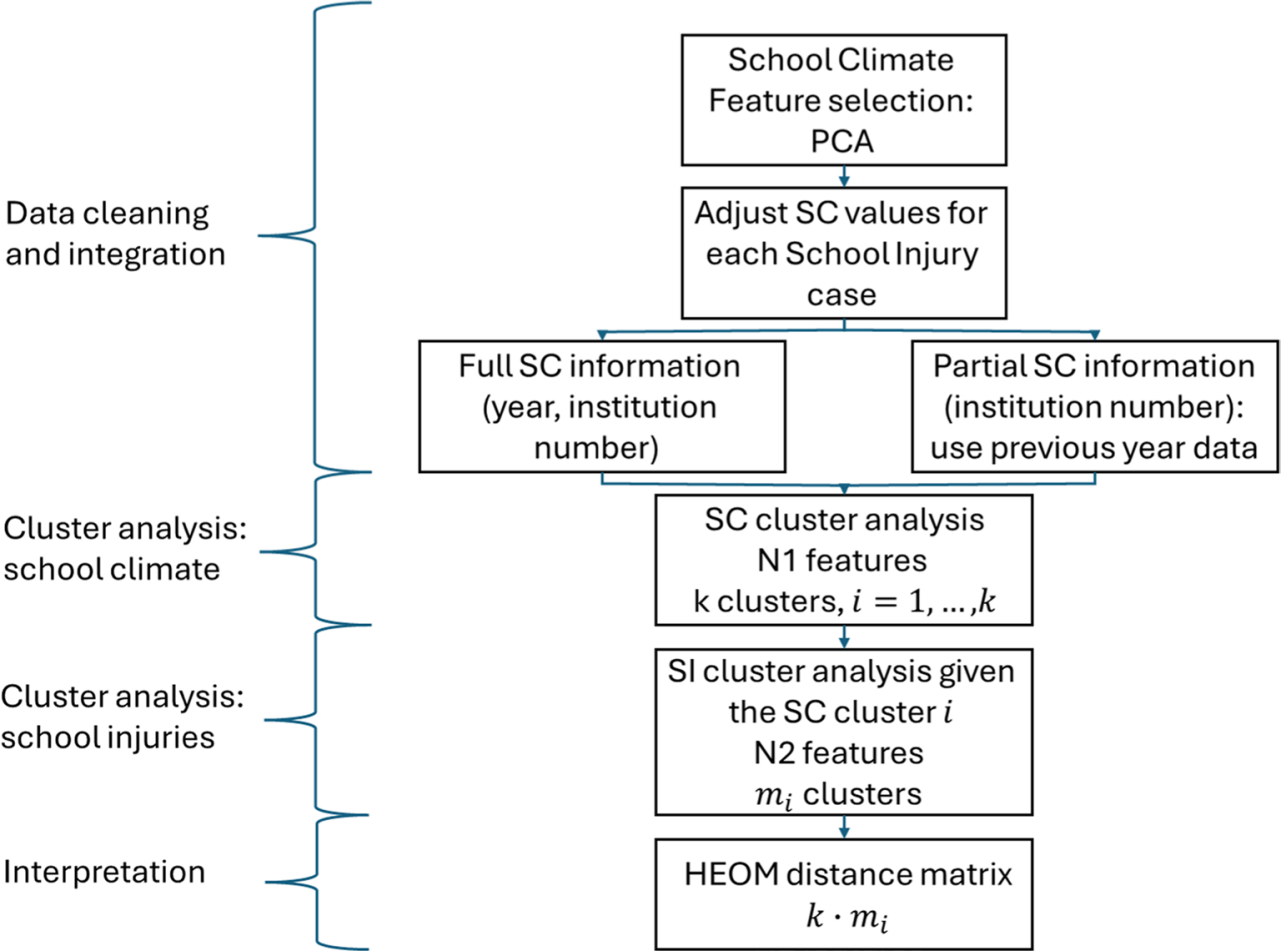
Research structure

The data analysis includes 4 main steps: (1) Analysis of variables distributions. Research data from both sources was analyzed using descriptive statistics (means, standard deviations, median values, Inter-Quartile Ranges and skewness metrics, Confidence Intervals (CI) (95%)) for numerical variables and frequencies distribution for categorical variables. (2) Cluster analysis of school climate variables using the k-means method. The clustering is performed per school. The analysis was performed using Weka software [10]. The number of clusters was set to five, this decision was dictated by the interpretation feasibility. In addition, the Schwarz's Bayesian Information Criteria (BIC) is used to justify the choice of final number of clusters. Each cluster is approximately defined as a "pattern" of school climate. The result shown for each cluster is the average vector of all variables across all included cases. It is called centroid. The clusters are analyzed using scatter plots. Each plot demonstrates the connection between one of the school climate variables and the "general feeling" variable, aggregated into five clusters. (3) Cluster analysis of all cases in the MDA dataset given the type of school climate found in step (2). The number of sub-clusters is defined as two to get the feasible interpretation of ten obtained clusters. (4) The distance between ten clusters is measured using the Heterogeneous Euclidean overlap metric (HEOM) implemented in Python [39]. The HEOM metric is useful in cases of mix data [9]. The distance between numerical variables is measured by normalized Euclidian distance, and between categorical valued by overlap metric 0/1. The metric is considered as robust and effective. The analysis is intended to quantitatively measure which clusters are close enough, so they can be defined as similar patterns. Figure 1 depicts the schematic structure of all research steps.

## Results

The Results chapter demonstrates four types of results, according to four steps presented in the Methods section. Starting with general data description, Table 1 displays the distribution of variables within two datasets.

**Table 1:** Variables distribution

Variable name	N of valid cases	Average (stdev)	CI (95%)	Median (IQR)	Skewness
Hour	10855	10.81 (1.493)	(10.7910.84)	11 (1012)	− 0.085
Age group	10363	8.92 (1.667)	(8.898.96)	9 (810)	− 0.226
N. of regular educ. classes	10853	15.077 (4.667)	(14.9915.16)	15 (1218)	0.15
N. of special educ. classes	10853	1.44 (1.437)	(1.421.47)	1 (02)	1.168
Average N. of students in regular educ. classes	10853	27.138 (3.694)	(27.0727.21)	27.17 (24.4429.6)	− 0.06
Average N. of students in special educ. classes	10853	28.695 (3.433)	(28.6328.76)	29.06 (26.3831.15)	− 0.308
Gender N (%)	N = 10585				
*boy*	7251 (69.3%)				
*girl*	3334 (30.7%)				
Event place N (%)	N = 10855				
*Classroom*	1662 (15.3%)				
*Corridor*	851 (7.8%)				
*Sportsground*	1341 (12.4%)				
*Sports hall*	390 (3.6%)				
*Stairs*	292 (2.7%)				
*Yard*	4216 (38.8%)				
*Otherwise*	2103 (19.3%)				
Event term N (%)	N = 10855				
*Before-after school*	284 (2.6%)				
*Break*	5146 (47.4%)				
*Lesson*	1479 (13.6%)				
*Sports class*	330 (3%)				
*Otherwise*	3616 (33.3%)				
Anatomic place main N (%)	N = 10855				
*Foot*	1810 (16.7%)				
*Hand*	2410 (22.2%)				
*Head*	5853 (53.9%)				
*Other*	782 (7.2%)				
Injury cause N (%)	N = 10855				
*Game*	4819 (44.4%)				
*Height*	354 (3.3%)				
*Slipping*	2534 (23.3%)				
*Violence*	579 (5.3%)				
*Otherwise*	2569 (23.7%)				
Injury type N (%)	N = 10852				
*Medium/deep incision*	52 (0.4%)				
*Superficial incision*	1433 (13.2%)				
*Light local burn*	12 (0.1%)				
*Rubbing tear*	707 (6.5%)				
*Trauma*	7608 (70.1%)				
*otherwise*	1040 (9.6%)				
Teachers and students' closeness	10766	67.07 (12.723)	(66.8367.31)	66.78 (58.6575.51)	0.208
Efforts involved	9648	62.533 (13.069)	(62.2762.79)	61.91 (54.1569.29)	0.299
Violence cases	10766	10.337 (3.975)	(10.2610.41)	10.42 (8.0612.63)	0.149
Teachers' satisfaction	10517	73.849 (9.238)	(73.6774.02)	75.4 (69.0580.12)	− 0.83
Parents involvement	9815	86.528 (7.29)	(86.3886.67)	87.5 (83.8291.67)	− 1.311
General positive feeling	10766	77.947 (10.352)	(77.7578.14)	77.56 (71.7584.41)	− 0.313

To summarize the most notable results from child injury data: the most frequent *event term* is break with 47.4%, the most frequent *event place* is yard with 38.8%, games with 44.4% of all injuries is the most prominent *injury cause*, and the most frequent injured *anatomic place* is head with 53.9%. Boys comprised around 70%, aged around 8.92 (median 9), particularly fourth or fifth graders.

The second step in the analysis is designed to divide all schools into different types of school climate "patterns". The selection of the number of clusters is based on two characteristics: the ability to interpret the obtained results and the measurement of Bayesian Information Criteria (BIC). The smaller the BIC value, the better is the segmentation. Here are the BIC values for the segmentation for 2–8 clusters: 27500 (2cl), 24380 (3cl), 21916 (4cl), 20293 (5cl), 18969 (6cl), 17867 (7cl), 17003 (8cl). After each iteration the possibility of reasonable interpretation was checked. This way we decided to stop at 5 clusters. The silhouette measures of cohesion and separation were about 0.45 (fair). Table 2 demonstrates five "patterns", according to five clusters. Importantly, cluster #3 emerges as a collection of schools with "good" climate, evidenced by high climate quality scores and low violence cases. In contrast, cluster #5 represents schools on the opposite end, marked by low scores and notable violence cases. Meanwhile, the remaining three clusters exhibit a certain alignment on the spectrum. The calculations of Z-scores for each value in the centroid strengthens the overall impression of distance between the obtained scale.

**Table 2:** Cluster analysis results – school climate (n. of cases, average, standard deviation and Z-score)

	Overall	Cluster 1	Cluster 2	Cluster 3	Cluster 4	Cluster 5
Number of cases in cluster	10855	3414	3210	1523	1865	843
Teachers and students' closeness	67.07 (12.72)	72.574 Z = 0.43	59.32 Z = − 0.61	87.489 Z = 1.61	63.047 Z = − 0.32	46.302 Z = − 1.63
Efforts involvement	62.533 (13.069)	65.733 Z = 0.24	56.787 Z = − 0.44	82.626 Z = 1.54	59.159 Z = − 0.26	42.616 Z = − 1.52
Violence cases	10.337 (3.975)	9.043 Z = − 0.32	11.858 Z = 0.38	5.096 Z = − 1.32	12.455 Z = 0.53	14.56 Z = 1.06
Teachers' satisfaction	73.849 (9.238)	77.386 Z = 0.38	76.064 Z = 0.24	80 Z = 0.66	60.428 Z = − 1.45	69.648 Z = − 0.45
Parents involvement	86.528 (7.29)	87.419 Z = 0.12	88.062 Z = 0.21	90.473 Z = 0.54	80.514 Z = − 0.82	83.265 Z = − 0.45
General positive feeling	77.947 (10.352)	82.741 Z = 0.46	72.409 Z = − 0.53	93.52 Z = 1.5	74.369 Z = − 0.35	59.4 Z = − 1.79

**Fig. 2:**
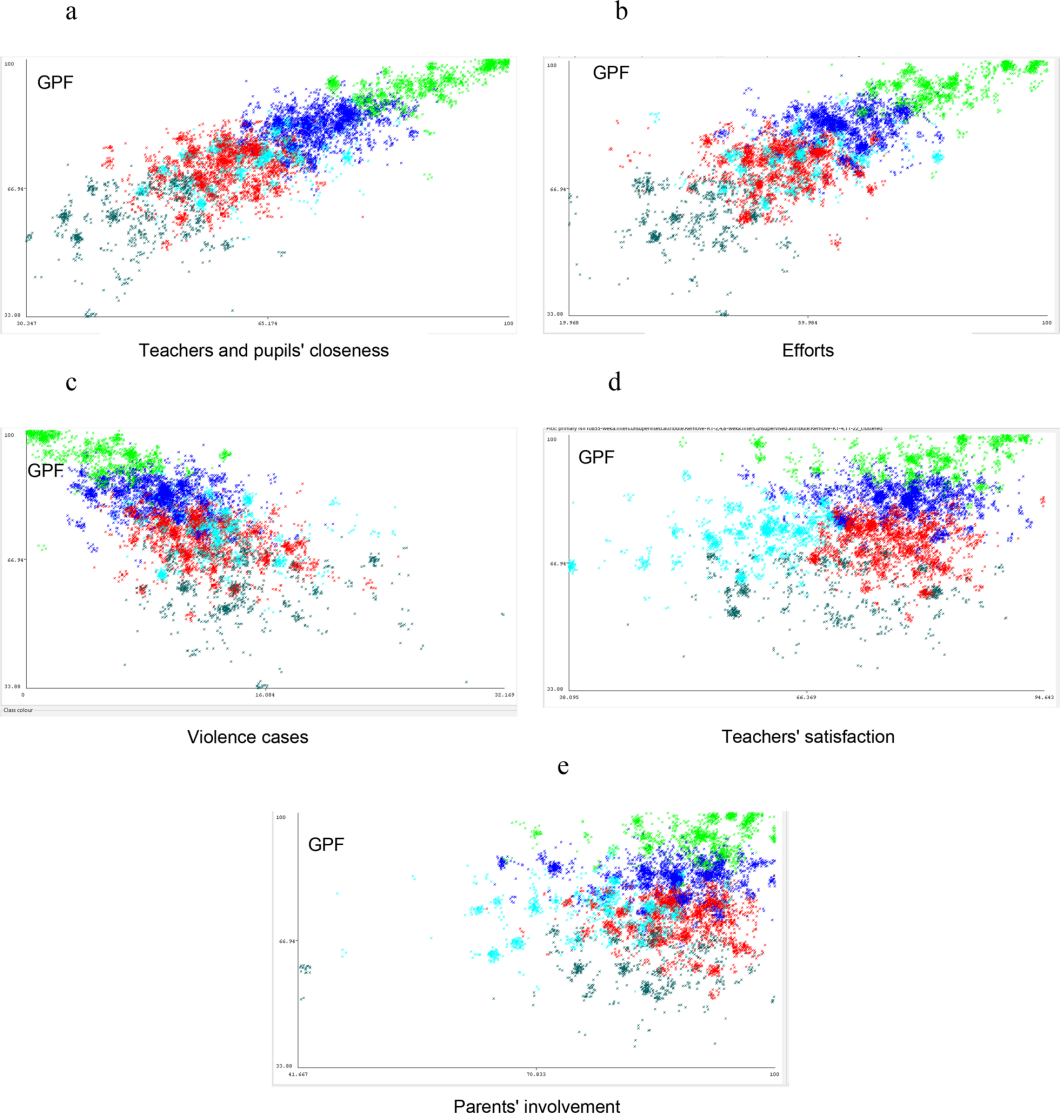
**a** Association between teachers and pupils' closeness and GPF; **b** Association between efforts and GPF; **c** Association between violence and GPF; **d** Association between teachers' satisfaction and GPF; **e** Association between parents' involvement and GPF

Figure 2a–e showcase scatter plots depicting cluster analysis outcomes from the first level of taxonomy. Each color in the scatter represents different cluster. These visual representations unveil intriguing patterns in the interplay between school climate factors and GPF. Figure 2a demonstrates positive correlation between *teacher-student closeness* and *GPF*. Clusters are distinguished by colors, aiding the categorization of diverse groups. Interestingly, clusters 2 (light blue) and 4 (red) display some overlap, diverging from the distinct separation seen in other clusters. This overlap signifies intricate dynamics within these clusters, suggesting complexities that merit deeper exploration and understanding. Figure 2b depicts the correlation between *efforts involved* and *GPF*, echoes the pattern observed in Fig. 2a. The positive correlation aligns with prior findings, indicating that increased engagement efforts enhance the overall positive atmosphere within the school. Figure 2c depicts the negative correlation between *violence instances* and *GPF*. Clusters 1, 2, and 4 intermingle, while clusters 3 and 5 stand distinctly apart. Figure 2d and e differ intriguingly from Fig. 2a–c. The correlations between *teachers' satisfaction*, *parental involvement,* and *GPF* (General Positive Feeling) remain unclear, as scattered data impedes cluster-specific identification.

Table 3 summarizes results from school injuries' cluster analysis. This phase follows the 1-st level of taxonomy, partitioning injury-related data within the five school climate clusters into two coherent sub-clusters. This uncovers patterns and relationships within and between sub-groups. Notably, the 1-st level of taxonomy unveiled clusters 1, 2, and 4 as in-between good (cluster 3) and bad (cluster 5) climates. We interpret the obtained sub-clusters in the 1-st cluster (in-between school climate), 3-rd cluster (good school climate), and 5-th cluster (good school climate).

**Table 3:** School injuries cluster analysis results

1-st level	Overall	Cluster 1 (N = 3414)		Cluster 2 (N = 3210)		Cluster 3 (N = 1523)		Cluster 4 (N = 1865)		Cluster 5 (N = 843)	
2-nd level		Cluster 1.1	Cluster 1.2	Cluster 2.1	Cluster 2.2	Cluster 3.1	Cluster 3.2	Cluster 4.1	Cluster 4.2	Cluster 5.1	Cluster 5.2
N. of cases	10855	900	2514	2055	1155	1136	387	713	1152	477	366
Age group	8.924	8.393	9.155	9.152	8.595	8.595	9.56	9.485	8.518	8.724	9.203
Gender											
*Boy*	7521 (69.3%)	247 (27.4%)	2115 (84.1%)	1452 (70.7%)	780 (67.5%)	965 (84.9%)	100 (25.8%)	486 (68.2%)	810 (70.3%)	318 (66.7%)	248 (67.8%)
*Girl*	3334 (30.7%)	653 (72.6%)	399 (15.9%)	603 (29.3%)	375 (32.5%)	171 (15.1%)	287 (74.2%)	227 (31.8%)	342 (29.7%)	159 (33.3%)	118 (32.2%)
Place event											
*Classroom*	1662 (15.3%)	321 (35.7%)	180 (7.2%)	263 (12.8%)	239 (20.7%)	158 (13.9%)	60 (15.5%)	118 (16.5%)	176 (15.3%)	59 (12.4%)	88 (24%)
*Corridor*	851 (7.8%)	107 (11.9%)	179 (7.1%)	149 (7.3%)	79 (6.8%)	86 (7.6%)	38 (9.8%)	46 (6.5%)	101 (8.8%)	40 (8.4%)	26 (7.1%)
*Sportsground*	1341 (12.4%)	55 (6.1%)	341 (13.6%)	318 (15.5%)	77 (6.7%)	174 (15.3%)	33 (8.5%)	91 (12.8%)	146 (12.7%)	68 (14.3%)	38 (10.4%)
*Sports hall*	390 (3.6%)	27 (3%)	100 (4%)	84 (4.1%)	39 (3.4%)	41 (3.6%)	24 (6.2%)	25 (3.5%)	24 (2.1%)	7 (1.5%)	19 (5.2%)
*Stairs*	292 (2.7%)	49 (5.4%)	53 (2.1%)	41 (2%)	39 (3.4%)	18 (1.6%)	20 (5.2%)	17 (2.4%)	33 (2.9%)	12 (2.5%)	10 (2.7%)
*Yard*	4216 (38.8%)	163 (18.1%)	1183 (47.1%)	1113 (54.2%)	139 (12%)	475 (41.8%)	130 (33.6%)	89 (12.5%)	613 (53.2%)	275 (57.7%)	36 (9.8%)
*Otherwise*	2103 (19.3%)	178 (19.7%)	478 (19%)	87 (4.2%)	543 (47.1%)	184 (16.2%)	82 (21.2%)	327 (45.8%)	59 (5.1%)	16 (3.3%)	149 (40.7%)
Event term											
*Before-after school*	284 (2.6%)	36 (4%)	68 (2.7%)	52 (2.5%)	31 (2.7%)	23 (2%)	10 (2.6%)	14 (2%)	30 (2.6%)	12 (2.5%)	8 (2.2%)
*Break*	5146 (47.4%)	365 (40.6%)	1263 (50.2%)	1374 (66.9%)	112 (9.7%)	574 (50.5%)	182 (47%)	47 (6.6%)	877 (76.1%)	325 (68.1%)	27 (7.4%)
*Lesson*	1479 (13.6%)	157 (17.4%)	262 (10.4%)	284 (13.8%)	183 (15.8%)	148 (13%)	64 (16.5%)	126 (17.7%)	129 (11.2%)	59 (12.4%)	67 (18.3%)
*Sports class*	330 (3%)	23 (2.6%)	89 (3.5%)	66 (3.2%)	18 (1.6%)	30 (2.6%)	14 (3.6%)	34 (4.8%)	27 (2.3%)	21 (4.4%)	8 (2.2%)
*Otherwise*	3616 (33.3%)	319 (35.4%)	832 (33.1%)	279 (13.6%)	811 (70.2%)	361 (31.8%)	117 (30.2%)	492 (69%)	89 (7.7%)	28 (5.9%)	256 (69.9%)
Anatomic place											
*Extremities*	4220 (38.9%)	381 (42.4%)	1003 (39.9%)	806 (39.3%)	412 (35.6%)	260 (22.9%)	290 (74.9%)	324 (45.4%)	384 (33.3%)	202 (42.3%)	158 (43.2%)
*Head*	5853 (53.9%)	471 (52.3%)	1322 (52.6%)	1076 (52.4%)	656 (56.8%)	805 (70.9%)	69 (17.8%)	338 (47.4%)	694 (60.2%)	247 (51.8%)	175 (47.8%)
*Other*	782 (7.2%)	48 (5.3%)	189 (7.5%)	173 (8.4%)	87 (7.5%)	71 (6.3%)	28 (7.2%)	51 (7.2%)	74 (6.4%)	28 (5.9%)	33 (9%)
Injury cause											
*Game*	4819 (44.4%)	96 (10.7%)	1373 (54.6%)	1112 (54.1%)	302 (26.1%)	650 (57.2%)	92 (23.8%)	339 (47.5%)	488 (42.4%)	269 (56.4%)	98 (26.8%)
*Falling from height*	354 (3.3%)	39 (4.3%)	75 (3%)	75 (3.6%)	42 (3.6%)	26 (2.3%)	10 (2.6%)	22 (3.1%)	38 (3.3%)	17 (3.6%)	10 (2.7%)
*Slipping*	2534 (23.3%)	448 (49.8%)	380 (15.1%)	230 (11.2%)	498 (43.1%)	173 (15.2%)	193 (49.9%)	167 (23.4%)	244 (21.2%)	98 (20.5%)	103 (28.1%)
*Violence*	579 (5.3%)	49 (5.4%)	118 (4.7%)	103 (5%)	67 (5.8%)	71 (6.3%)	8 (2.1%)	59 (8.3%)	67 (5.8%)	18 (3.8%)	19 (5.2%)
*Otherwise*	2569 (23.7%)	268 (29.8%)	568 (22.6%)	535 (26%)	246 (21.3%)	216 (19.1%)	84 (21.8%)	126 (17.6%)	315 (27.2%)	75 (15.7%)	136 (37.1%)
Injury type											
*Medium/deep incision*	52 (0.4%)	4 (0.4%)	11 (0.5%)	5 (0.2%)	9 (0.8%)	11 (0.9%)	2 (0.5%)	1 (0.1%)	5 (0.5%)	3 (0.6%)	1 (0.3%)
*Superficial incision*	1433 (13.2%)	146 (16.2%)	278 (11.1%)	235 (11.4%)	175 (15.2%)	192 (16.9%)	32 (8.3%)	88 (12.3%)	148 (12.8%)	79 (16.6%)	60 (16.4%)
*Rubbing tear*	707 (6.5%)	54 (6%)	179 (7.1%)	143 (7%)	75 (6.5%)	74 (6.5%)	18 (4.7%)	40 (5.6%)	83 (7.2%)	23 (4.8%)	18 (4.9%)
*Trauma*	7368 (67.9%)	54 (6%)	1784 (71%)	1419 (69%)	835 (72.3%)	776 (68.3%)	293 (75.7%)	537 (75.3%)	769 (66.8%)	333 (69.9%)	245 (66.9%)
*otherwise*	1295 (12%)	79 (8.7%)	262 (10.3%)	253 (12.3%)	61 (5.3%)	83 (7.3%)	41 (10.7%)	46 (6.5%)	146 (12.7%)	39 (8.2%)	42 (11.5%)

Clusters 1.1 and 1.2 notably diverge in gender composition. Cluster 1.1 consists mostly of girls (72.6%), with 35.7% of injuries in classrooms and 18.1% in the school yard. During breaks, 40.6% of incidents occurred, with 49.8% slipping incidents and 10.7% playing incidents. Meanwhile, Cluster 1.2 is predominantly boys (84.1%), with 47.1%-yard injuries, 50.2% during breaks, and 10.4% during classes. A significant 54.4% occurred during games, 15.1% due to slipping. These disparities emphasize the uniqueness of both clusters' characteristics. Cluster 3, symbolizing "good" climate, splits into two sub-clusters: 3.1 with 1136 injuries and 3.2 with 387. Notably, Cluster 3.1 has 87.9% boys, Cluster 3.2, 74.2% girls. In 3.1, 41.8% injuries occur in yards, 15.3% on sports fields, 13.9% in classrooms. In 3.2, 33.6% injuries are in yards, 15.5% in classrooms, 9.8% in corridors. Syncing with injury timing, 50.5% in 3.1 and 47% in 3.2 happened during breaks. Anatomical injury distribution varies: 70.9% head injuries in 3.1, 75% limb damage in 3.2. These intricacies intertwine with incident context: 57.2% game-related injuries in 3.1, 49.9% due to slipping in 3.2. Such complexity enriches the context of Clusters 3.1 and 3.2 in a good school climate. Finally, we explore sub-clusters denoting a bad school climate, as seen in Cluster 5. In both sub-clusters, around two-thirds are boys. Cluster 5.1, with 477 cases, records 57.7% incidents in yards, and 68.1% during breaks. Notably, 56.4% incidents coincide with recreational activities. Contrastingly, Cluster 5.2 reveals a varied pattern. Here, 24% incidents arise in classrooms, with just 9.8% in yards. Effective interpretation is problematic due to unreported injury event term (69.9%). This cluster contained a substantial amount of missing information, meaning that any conclusions drawn from interpreting the results should be viewed with caution. Alternatively, one could assume a uniform distribution of the other possibilities across the total number of subjects. Incidents differ: 26.8% during games, 28.1% linked to slipping. These complexities illuminate the multifaceted attributes of adverse climate sub-clusters, deepening our comprehension of the intricate interaction between school environment and injuries.

Table 4 introduces a heatmap illustrating the distance matrix between all ten clusters. Employing a specialized index for mixed data, Heterogeneous Euclidean overlap metric (HEOM), we constructed a matrix to compare all 10 clusters formed during analysis. Each intersection represents a distance measure, indicating distance between clusters. Cluster 3.2 stands out, mainly girls in favorable climate schools with limb injuries, notably distant from all clusters. This underlines its distinctiveness, accentuating unique attributes within the dataset.

**Table 4:**
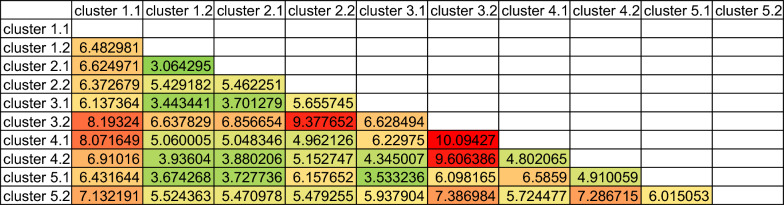
Distance matrix of Heterogeneous Euclidean overlap metric between obtained clusters

The high distance value of 10.0427 indicates significant dissimilarity between clusters 3.2 and 4.1. A similarly high value of 9.6064 between clusters 3.2 and 4.2 suggests notable differences. These distances likely reflect distinct injury characteristics prevalent in clusters predominantly comprising girls compared to others. Notably, Cluster 3.2, with 74.2% girls, shows marked differences from most other clusters. The lowest distance values are observed between Cluster 1.2 and other clusters, as well as between Cluster 2.1 and other clusters. These findings can be readily explained by the predominance of injuries occurring during breaks, most frequently involving head injuries. As highlighted in our previous article, this type of injury is the most common [18].

## Discussion

This study delves into the complex interaction between school climate and child injuries in primary schools. The intricate nature of this phenomenon demands advanced analytic approaches. Traditional methods may fall short, prompting the recommendation of techniques like multivariate analysis, machine learning, and data mining techniques to unveil hidden patterns. The present article introduces methodological innovations on multiple fronts. Firstly, it employs a two-dimensional cluster analysis, each dimension drawing from distinct databases. Secondly, it pioneers the application of this methodology in previously unexplored domains such as school climate and school injury data. Lastly, it introduces a novel practice by cross-referencing information from disparate sources.

Constructing indices and collecting data about factors of school climate and school injuries can indeed provide a multidimensional view of the educational environment and the circumstances that may be associated with school injuries [32]. However, extracting meaningful insights from this information and translating them into effective decision-making is not always straightforward, and there can be challenges in aligning the different dimensions of data and motivations behind their use [6]. Statistical methods facilitate comparisons, yet an expert's interpretation, fused with quantitative outcomes, can enhance the interpretation's depth. This blending of logical reasoning and data-driven results enriches comprehension, addressing potential limitations of purely statistical approaches [8].

The proposed 2-levels taxonomy categorizes cases into distinct subgroups based on their similarities/dissimilarities using cluster analysis methodology. The anticipated relationship between school injuries and climate characteristics reached an impasse, prompting the adoption of machine learning for an unbiased, unsupervised taxonomy. Initially, school climate events formed five clusters, representing diverse climate patterns, ranging from "good" to "bad". In the 2-nd phase of the taxonomy, each "pattern" was subdivided into two sub-clusters, this time using the injury variables. This approach provides a nuanced understanding of complex interactions within primary school environments. Remarkably, a profound insight emerges from analyzing distinctions among sub-clusters within the initial taxonomy's five clusters. Not surprisingly, the quality of school climate—whether good, bad, or in-between—has very complex effect on the characteristics of school injuries.

By taking a systematic and data-driven approach to understanding and preventing injuries, one can create a safer school environment for students and reduce the potential injuries associated with specific patterns in the school climate. A wide variety of tailored (precision) measures can be used by decision makers at all levels—from teachers, through school managers, to government or local authorities. Moving beyond apparent patterns, the exploration of injury characteristics, along with class size, regular and special education classes, and school climate, has the potential to verify or question existing hypotheses relevant to decision-makers.

### Implications for school health policy

The study reveals a complex relationship between school climate and school injuries, highlighting the need for advanced analysis methods beyond traditional approaches. The study's dataset, drawn from unrelated sources, underscores the interdisciplinary nature of school health. The taxonomy exposes the multifaceted nature of school environments, urging tailored policies for injury prevention. Notably, insights challenge assumptions about climate-injury links, guiding strategic decision-making. The study bridges qualitative insights and quantitative analysis, yielding comprehensive interpretations. Below are several recommendations for implementation by the decision makers in the health care system and the education system.

(1) **Implementation of Targeted Safety Measures and Training Programs**. Educational authorities must develop and implement targeted safety training programs that cater to the specific injury patterns observed in different clusters. For example, for Cluster 1.1, where slipping incidents are prevalent, schools should install anti-slip flooring and conduct regular maintenance checks. For Cluster 1.2, where injuries during games are common, structured play and supervised sports activities should be introduced to minimize risks. We recommend installing non-slip surfaces in playgrounds, establishing designated safety zones, and placing surveillance cameras near playground areas to enhance student safety. Health system authorities must collaborate with schools to provide first-aid training for teachers and staff, ensuring they can promptly and effectively respond to injuries. Additionally, establish a reporting system for injuries to track and analyze incident patterns, enabling continuous improvement of safety measures. (2) **Promotion of Gender-Sensitive Safety Interventions**. We recommend to educational system decision-makers to recognize and address the gender-specific injury patterns by designing gender-sensitive safety interventions. For instance, since girls in Cluster 1.1 experience more injuries in classrooms, schools should evaluate classroom layouts and introduce safer furniture and equipment. For boys in Cluster 1.2, interventions could focus on safer playground designs and the introduction of rules that promote safe play during breaks. In addition, we recommend to the health system decision-makers to provide resources for gender-specific health education programs that raise awareness about common injury risks and prevention strategies among students. Health professionals can collaborate with schools to develop educational materials and workshops tailored to the different needs of boys and girls. (3) **Enhancement of School Climate and Environment**. We suggest improving overall school climate by fostering a supportive and safe environment. For Clusters 3.1 and 3.2, with good climates but varying injury contexts, schools should encourage positive behavior and peer support systems to mitigate risky behaviors during breaks. In schools with bad climates (Cluster 5), initiatives to improve school climate could include conflict resolution programs, anti-bullying campaigns, and increased supervision during high-risk periods such as breaks and recreational activities. We suggest implementing social-emotional learning programs and creating a more inclusive environment. If the health system organizations will work with educational institutions to conduct regular assessments of school climate and its impact on student health and safety, it can either improve the overall situation.

### Limitations and future research

The reliability and accuracy of the data from different sources could questionable, affecting the robustness of the findings. The study's findings may be specific to the context of Israeli primary schools and may not be applicable universally. The study is mainly methodological. A notable disparity could exist between the envisioned outcome and the actual findings in a particular application. Despite advanced analysis, the interpretation of cluster results and relationships still involves some level of subjectivity, potentially impacting the conclusions. The study uses data from a specific timeframe, and changes over time might not be adequately captured, limiting the study's temporal validity. There are possibly injury cases that handled in the school by the educational/administrative staff. We used only cases that were reported to MDA. While data from multiple sources enriches the analysis, challenges related to merging datasets may introduce errors or inconsistencies. More comprehensive information could significantly contribute to advancing research in this field. The distinctive characteristics of populations in Israel, including the multicultural composition of specific geographic regions and the socio-economic disparities between various societal groups, offer a valuable foundation for deeper exploration. These factors could help frame future studies and shed light on how diverse school environments and demographic variations influence school injuries.

## Conclusions

The study demonstrates how advanced analytic techniques such as multivariate analysis and machine learning can uncover intricate relationships between school climate and child injuries in primary schools, overcoming limitations of traditional methods. By employing a two-dimensional cluster analysis that draws data from separate databases, the study innovatively integrates disparate sources, providing a multidimensional perspective on how various aspects of school climate might correlate with injury incidences. This approach, combined with the novel practice of cross-referencing data, enables a nuanced understanding of the school environment and supports more informed decision-making aimed at improving student safety and reducing injuries. The creation of a taxonomy that categorizes school climate and injury patterns into distinct subgroups further enhances this understanding, revealing complex interactions and allowing for the development of tailored preventive measures.

## Data Availability

The datasets are not publicly available.

## Abbreviations

MDA: Magen david adom
EMS: Emergency medical services
CI: Confidence interval
IQR: Inter-quartile range
HEOM: Heterogeneous euclidean overlap metric
GPF: General positive feeling

## References

[1] Aldridge JM, McChesney K. The relationships between school climate and adolescent mental health and wellbeing: a systematic literature review. Int J Educ Res. 2018;88:121–45. 10.1016/j.ijer.2018.01.012.

[2] Anglin JP. Risk, well-being, and paramountcy in child protection: the need for transformation. Child Youth Care Forum. 2002;31(4):233–55. 10.1023/A:1016303309618.

[3] Benassi M, Garofalo S, Ambrosini F, Sant’Angelo RP, Raggini R, De Paoli G, Ravani C, Giovagnoli S, Orsoni M, Piraccini G. Using two-step cluster analysis and latent class cluster analysis to classify the cognitive heterogeneity of cross-diagnostic psychiatric inpatients. Front Psychol. 2020. 10.3389/fpsyg.2020.01085. 32587546 10.3389/fpsyg.2020.01085 PMC7299079

[4] Caruso G, Gattone SA, Fortuna F, Di Battista T. Cluster analysis as a decision-making tool: a methodological review. In: Bucciarelli E, Chen S-H, Corchado JM, editors. Decision economics In: the tradition of herbert a. Simon’s Heritage. Berlin: Springer; 2018. p. 48–55. 10.1007/978-3-319-60882-2_6.

[5] Cavrini G, Chianese G, Bocch B, Dozza L. School climate: parents’, students’ and teachers’ perceptions. Procedia Soc Behav Sci. 2015;191:2044–8. 10.1016/j.sbspro.2015.04.641.

[6] Centers for Disease Control and Prevention, USA. School health guidelines to prevent unintentional injuries and violence. *MMWR: Recommendations and Reports: Morbidity and Mortality Weekly Report. Recommendations and Reports / Centers for Disease Control*, *50*(RR-22), 2001; 1–73. 11770577

[7] Derosier ME, Newcity J. Students’ perceptions of the school climate. J Sch Violence. 2005;4(3):3–19. 10.1300/J202v04n03_02.

[8] Esnard A-M, Lai B. Interdisciplinary approaches to examining postdisaster school recovery. Risk Anal. 2021;41(7):1227–31. 10.1111/risa.13137. 29989188 10.1111/risa.13137

[9] Foss AH, Markatou M, Ray B. Distance metrics and clustering methods for mixed-type data. Int Stat Rev. 2019;87(1):80–109. 10.1111/insr.12274.

[10] Frank E, Hall MA, Witten IH. The WEKA workbench. San Francisco: Morgan Kaufmann; 2016.

[11] Furlong MJ, Greif JL, Bates MP, Whipple AD, Jimenez TC, Morrison R. Development of the California school climate and safety survey-short form. Psychol Sch. 2005;42(2):137–49. 10.1002/pits.20053.

[12] Gore GC, Magdalinos H, Pless IB. School injuries and preventive policies and programs. Can J Public Health. 2004;95(6):424–8. 10.1007/BF03403986. 15622790 10.1007/BF03403986 PMC6975750

[13] Gottfredson GD, Gottfredson DC. What schools do to prevent problem behavior and promote safe environments. J Educ Psychol Consult. 2001;12(4):313–44. 10.1207/S1532768XJEPC1204_02.

[14] Gratz RR. School injuries: what we know, what we need. J Pediatric Health Care. 1992;6:256–62. 10.1016/0891-5245(92)90024-X. 10.1016/0891-5245(92)90024-x 1403570

[15] Grira N, Crucianu M, Boujemaa N. Unsupervised and semi-supervised clustering: a brief survey. Rev Machine Learn Techn Process Multimed Content. 2005;1(2004):9–16.

[16] Holland W, Mossialos E, Merkel B. Public health policies in the European union. Abingdon: Routledge; 2018.

[17] Jaffe DH, Goldman S, Peleg K, The Israel Trauma Group. The role of community in pediatric injury. J Commun Health. 2011;36(2):244–52. 10.1007/s10900-010-9304-z. 10.1007/s10900-010-9304-z 20945082

[18] Jaffe E, Khalemsky A, Khalemsky M. Game-related injuries in schools: a retrospective nationwide 6-year evaluation and implications for prevention policy. Israel J Health Policy Res. 2021;10(1):51. 10.1186/s13584-021-00487-5. 10.1186/s13584-021-00487-5 PMC8404308 34461983

[19] Kraus R, Horas U, Szalay G, Alt V, Kaiser M, Schnettler R. School-related injuries: a retrospective 5-year evaluation. Eur J Trauma Emerg Surg. 2011;37(4):411–8. 10.1007/s00068-010-0063-4. 26815278 10.1007/s00068-010-0063-4

[20] Kutsyuruba B, Klinger DA, Hussain A. Relationships among school climate, school safety, and student achievement and well-being: a review of the literature. Rev Edu. 2015;3(2):103–35. 10.1002/rev3.3043.

[21] Laflamme L, Menckel E. Pupil injury risks as a function of physical and psychosocial environmental problems experienced at school. Inj Prev. 2001;7(2):146–9. 10.1136/ip.7.2.146. 11428563 10.1136/ip.7.2.146 PMC1730719

[22] Lee T, Cornell D, Gregory A, Fan X. High suspension schools and dropout rates for black and white students. Educ Treat Child. 2011;34(2):167–92.

[23] MacKay M. Playground injuries. Inj Prev. 2003;9(3):194–6. 10.1136/ip.9.3.194. 12966004 10.1136/ip.9.3.194 PMC1730990

[24] McEvoy P, Richards D. A critical realist rationale for using a combination of quantitative and qualitative methods. J Res Nurs. 2006;11(1):66–78. 10.1177/1744987106060192.

[25] McGiboney GW. The psychology of school climate. Cambridge: Cambridge Scholars Publishing; 2016.

[26] McKee M, Zwi A, Koupilova I, Sethi D, Leon D. Health policy-making in central and eastern Europe: lessons from the inaction on injuries? Health Policy Plan. 2000;15(3):263–9. 10.1093/heapol/15.3.263. 11012400 10.1093/heapol/15.3.263

[27] Meraviglia MG, Becker H, Rosenbluth B, Sanchez E, Robertson T. The expect respect project. Creating a positive elementary school climate. J Interpersonal Violence. 2003;18(11):1347–60. 10.1177/0886260503257457. 10.1177/0886260503257457 19774770

[28] Mitchell MM, Bradshaw CP, Leaf PJ. Student and teacher perceptions of school climate: a multilevel exploration of patterns of discrepancy. J Sch Health. 2010;80(6):271–9. 10.1111/j.1746-1561.2010.00501.x. 20573139 10.1111/j.1746-1561.2010.00501.x

[29] Morrongiello BA, Schell SL. Child injury: the role of supervision in prevention. Am J Lifestyle Med. 2010;4(1):65–74. 10.1177/1559827609348475.

[30] Mytton J, Towner E, Brussoni M, Gray S. Unintentional injuries in school-aged children and adolescents: lessons from a systematic review of cohort studies. Inj Prev. 2009;15(2):111–24. 10.1136/ip.2008.019471. 19346424 10.1136/ip.2008.019471

[31] Ramsey CM, Spira AP, Parisi JM, Rebok GW. School climate: perceptual differences between students, parents, and school staff. Sch Eff Sch Improv. 2016;27(4):629–41. 10.1080/09243453.2016.1199436. 28642631 10.1080/09243453.2016.1199436 PMC5473611

[32] Sethi D, Europe, W. H. O. R. O. for. (2008). *European Report on Child Injury Prevention*. WHO Regional Office Europe.

[33] Street EJ, Jacobsen KH. prevalence of sports injuries among 13- to 15-year-old students in 25 low- and middle-income countries. J Commun Health. 2017;42(2):295–302. 10.1007/s10900-016-0255-x. 10.1007/s10900-016-0255-x 27639867

[34] Thapa A, Cohen J, Higgins-D’Alessandro A, Guffey S. School Climate Research Summary: August 2012. School Climate Brief, Number 3. In *National School Climate Center*. National School Climate Center. 2012. https://eric.ed.gov/?id=ED573683

[35] *Unit 7: Injury Prevention and Safety Promotion | Healthy Schools | CDC*. 2023. https://www.cdc.gov/healthyschools/bam/injury.htm

[36] Wang C, Berry B, Swearer SM. The critical role of school climate in effective bullying prevention. Theory Into Practice. 2013;52(4):296–302. 10.1080/00405841.2013.829735.

[37] Welsh WN. The effects of school climate on school disorder. Ann Am Acad Pol Soc Sci. 2000;567:88–107.

[38] *WHO Health Emergency Appeal*. (n.d.). WHO Health Emergency Appeal. Retrieved May 16, 2023, from https://healthemergencyappeal.who.foundation/

[39] Wilson DR, Martinez TR. Improved heterogeneous distance functions. J Artif Intell Res. 1997;6:1–34. 10.1613/jair.346.

